# Chimeric models of neurodegenerative diseases: from disease mechanisms to regenerative therapies

**DOI:** 10.1186/s44477-026-00059-4

**Published:** 2026-11-10

**Authors:** Laura Cáceres-Palomo, María Alfonso-Triguero, Arantzazu Belloso-Iguerategui, Estibaliz Santiago-Mujika, Marta Mirón-Alcalá, Amaia M. Arranz

**Affiliations:** 1 Achucarro Basque Center for Neuroscience, Leioa, Spain; 2 Department of Neurosciences, University of the Basque Country (EHU), Leioa, Spain; 3 IKERBASQUE Basque Foundation for Science, Bilbao, Spain

**Keywords:** Human-rodent chimeras, Xenotransplantation, Human pluripotent stem cells, Neurodegenerative diseases, Glia, Cell replacement therapy

## Abstract

Human-rodent chimeric CNS models provide a unique platform for investigating human-specific aspects of neurodegeneration within the physiological environment of the living CNS. These models are generated by transplanting human pluripotent stem cell-derived neurons, astrocytes, microglia, oligodendrocytes, or neural or glial progenitor cells into the CNS of permissive host animals. Following transplantation, human cells mature, functionally integrate within host tissue, and retain key species-specific molecular and cellular features. Here, we review recent advances in the use of chimeric models to investigate disease mechanisms in Alzheimer's disease, Parkinson's disease, Huntington's disease, Amyotrophic Lateral Sclerosis, and demyelinating disorders. We highlight the unique insights these models have provided into human-specific cellular vulnerabilities, genotype-dependent phenotypes, glial responses, and neuroimmune interactions *in vivo*. We also discuss emerging translational applications, including cell replacement therapies, engineered glial delivery platforms, and remyelination strategies, some of which have progressed toward clinical translation. Finally, we outline the current limitations and future directions for developing next-generation multi-lineage humanized models with enhanced disease fidelity and translational potential.

## Introduction

In classical mythology, the Chimera was a fearsome fire-breathing monster—part lion, part goat, part serpent—an entity so unnatural that it came to embody the impossible [1]. For centuries, the term retained this connotation: a chimera was something one could imagine but never realize. In modern biology, however, chimeric systems have overturned this meaning. What once symbolized the impossible has become a powerful experimental tool in neuroscience.

Alzheimer's disease (AD), Parkinson's disease (PD), Amyotrophic Lateral Sclerosis (ALS), Huntington's disease (HD), and demyelinating disorders collectively represent major challenges in contemporary biomedicine, affecting hundreds of millions of individuals worldwide with prevalence expected to rise sharply as populations age [2]. Despite decades of intensive research, disease-modifying therapies remain unavailable or limited for the vast majority of these conditions [3]. This reflects a major gap: the biological mechanisms that drive neurodegeneration remain incompletely understood. Neurodegeneration is a multifactorial process, arising from the convergence of genetic risk, aging, environmental insults, and molecular events [3]. Moreover, its defining hallmarks, including aberrant protein aggregation, neuroinflammation, synaptic dysfunction, and selective neuron and glia vulnerability, emerge from complex interactions between diverse cell types within living neural circuits [4, 5].

Over the past decades, a broad repertoire of experimental models has transformed our understanding of these disorders. Transgenic rodent models have been indispensable for investigating disease mechanisms *in vivo* and for preclinical therapeutic testing. Likewise, human pluripotent stem cell (hPSC)-based platforms, including two-dimensional (2D) cultures and three-dimensional (3D) brain organoids, have provided access to patient-derived human neural cells, revealing cell-type-specific vulnerabilities, validating genetic risk factors, and providing scalable platforms for drug discovery [6–8]. More recent human-rodent chimeric models bridge these complementary experimental systems by combining the molecular identity of human cells with the physiological complexity of the living mammalian central nervous system (CNS). Human brain cells transplanted into the rodent CNS mature, functionally integrate into host neural circuits, and interact with resident neurons, glia, and vascular cells while preserving key features of their human-specific biology [9, 10]. Although most studies to date have focused on the brain, human cell engraftment and integration can also extend to other regions of the CNS, including the spinal cord, an important consideration for disorders in which spinal pathology is a prominent component, such as ALS, MS, and other demyelinating diseases. This combination provides an experimental framework for investigating human-specific disease mechanisms *in vivo*. For example, human neurons transplanted into murine AD models develop tau pathology and undergo neurodegeneration while adjacent mouse neurons remain unaffected [11, 12]; human dopaminergic neurons derived from PD patients exhibit disease-associated pathological features that are absent in neighboring murine neurons [13, 14]; human glial progenitor cells carrying HD mutations display myelination defects not observed in rodent glia [15], and transplanted human oligodendrocyte progenitors have uncovered species-specific mechanisms of myelin formation and repair in demyelinating disease models. Beyond disease modeling, chimeric systems also provide preclinical platforms for evaluating regenerative strategies, including cell replacement therapies, by enabling assessment of human cell survival, maturation, functional integration, and therapeutic efficacy within the living CNS.

**Fig. 1:**
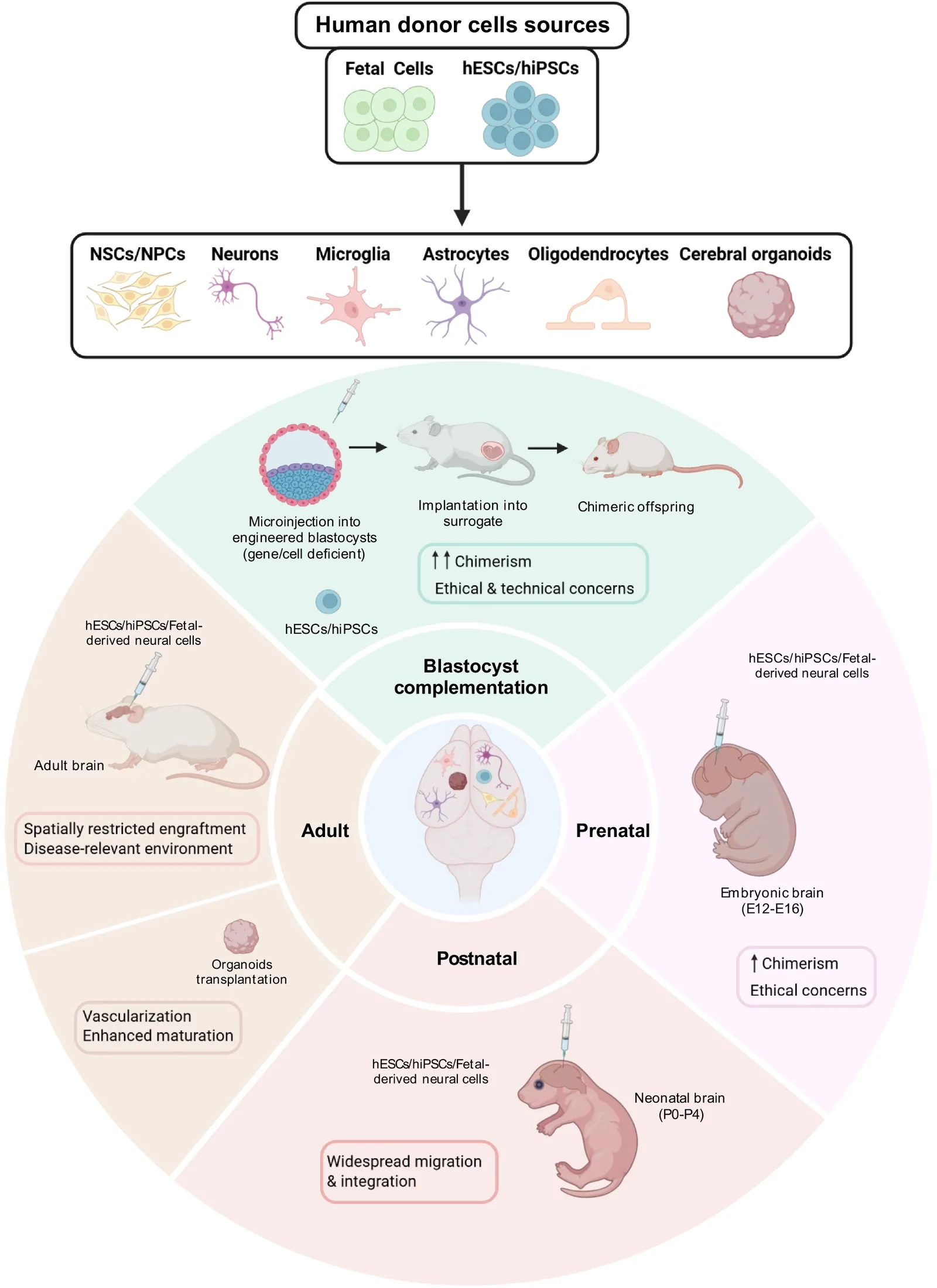
**Overview of the major approaches used to generate neural chimeric models.** Human neural chimeras can be generated using complementary approaches that differ in the developmental stage of the host and the nature of the donor material. Human embryonic stem cells (hESCs), human induced pluripotent stem cells (hiPSCs), or fetal-derived neural cells can be introduced into genetically engineered blastocysts through blastocyst complementation, or transplanted into the prenatal (E12–E16), neonatal (P0–P4), or adult brain. Alternatively, human brain organoids can be engrafted into the adult brain. The choice of transplantation strategy influences donor cell engraftment, migration, maturation, and integration, as well as the experimental applications of the resulting chimeric models. *Created with BioRender*

Human-rodent neural chimeras can be generated through several complementary approaches that differ in the developmental stage of the host and the nature of the transplanted human material (Fig. 1). These include blastocyst complementation and transplantation of human cells during prenatal, neonatal, or adult stages, as well as xenotransplantation of human brain organoids. Developmental transplantation generally provides a more permissive environment for widespread migration, maturation, and integration of human cells, whereas transplantation into the adult CNS typically results in more spatially restricted engraftment but enables human cells to be studied within mature, diseased, or injured tissue. Organoid xenotransplantation provides an additional strategy in which human neural tissue can become vascularized, mature, and establish functional connections with the host *in vivo*. The methodological principles, advantages, and limitations of these approaches have been comprehensively reviewed [10].

In this review, we examine the use of CNS chimeric models to investigate the mechanisms underlying neurodegeneration and to develop therapeutic strategies. We discuss how these models have advanced our understanding of AD, PD, HD, ALS, and demyelinating disorders, with particular emphasis on the human-specific cellular and molecular features they reveal. Within each disease context, we distinguish their use as experimental models for investigating human-specific disease mechanisms from xenotransplantation approaches primarily aimed at evaluating cell replacement and regenerative strategies. Finally, we discuss the current limitations and future directions of the field including improving chimerism efficiency, incorporating adaptive immunity, and generating increasingly complex multi-lineage humanized CNS models; as well as the potential of these approaches to advance regenerative and cell-based therapies.

## Chimeric models of Alzheimer’s disease

AD is the leading cause of dementia, neuropathologically characterized by amyloid-β (Aβ) plaques, tau pathology, neuroinflammation, vascular alterations, synaptic dysfunction, and progressive neuronal loss. AD is now widely recognized as a multicellular disorder involving astrocytes, microglia, oligodendrocytes, vascular cells and neurons, all of which contribute to disease onset and progression [5, 16]. To model AD, researchers have traditionally relied on transgenic mouse lines overexpressing mutant forms of human *APP* and/or *PSEN1*, including APP/PS1, 3xTg-AD and 5xFAD. More recently, knock-in models, such as *App* NL-G-F, have been developed by introducing humanized familial AD mutations into the endogenous mouse *App* locus without transgene overexpression [17, 18]. Building upon these experimental systems, human-rodent chimeric models have emerged as a powerful platform for investigating human neurons and glial cells within the multicellular and physiological environment of the diseased brain. By enabling direct assessment of how human neurons and glial cells respond to amyloid deposition, tau pathology, and the inflammatory brain microenvironment, these models have uncovered human-specific cellular vulnerabilities, the functional consequences of genetic risk variants, and molecular mechanisms that are difficult to recapitulate in conventional mouse models. In addition, they provide a valuable preclinical platform for evaluating cell-based therapies and other therapeutic strategies. In the following sections, we discuss how chimeric models incorporating distinct human brain cell types have advanced our understanding of AD pathogenesis while highlighting their emerging applications for therapeutic development.

### Neuron AD chimeras

Because neuronal degeneration is the ultimate pathological hallmark of AD, the first human-rodent chimeric models were developed to determine whether human neurons exhibit intrinsic susceptibility to AD pathology, and to investigate how AD-associated genetic variants influence neuronal vulnerability. Human embryonic stem cells (hESCs) and human induced pluripotent stem cells (hiPSCs) derived from healthy individuals or AD patients have been differentiated into neural progenitor cells (hNPCs) or neurons and transplanted into the forebrain of amyloid-producing mouse models. These studies have primarily employed transgenic APP/PS1 mice as well as *App* NL-G-F knock-in models crossed with immunodeficient NOD-SCID or Rag2 knockout mice. In some cases, tau pathology has been further enhanced using transgenic tau models, such as PS19 (P301S), either alone or in combination with amyloid models.

The first demonstration that transplanted human neurons develop AD pathology *in vivo* came from Espuny-Camacho and colleagues, who showed that human neurons transplanted into neonatal AD mice integrated into host circuitry and, six months after transplantation, developed hallmark AD features including neuritic dystrophy, dendritic spine loss, tau hyperphosphorylation and neuronal degeneration, whereas neighboring mouse neurons remained largely unaffected [11]. Building upon these findings, Balusu and colleagues demonstrated progressive development of tau pathology and granulovacuolar degeneration in transplanted human neurons, while identifying upregulation of the long non-coding RNA *MEG3* and activation of the RIPK1-RIPK3-MLKL necroptosis pathway as a human-specific mechanism of neuronal death [12]. Together, these studies illustrate a key strength of chimeric models: human and mouse neurons can be exposed to the same pathological environment *in vivo*, allowing species-specific and cell-intrinsic vulnerabilities to be identified. The selective degeneration of human neurons indicates that the response to amyloid pathology differs substantially between human and murine neurons.

Beyond revealing species-specific neuronal vulnerability, chimeric models enable interrogation of the effects of human AD-associated genetic variants *in vivo*, within a common pathological environment. Following transplantation into *APOE* knock-in mice, human neurons carrying the *APOE4* allele displayed increased vulnerability, p53- and senescence-associated transcriptional programs, selective loss of inhibitory neurons, and enhanced Aβ aggregation compared with *APOE3* neurons [19]. Depletion of host microglia markedly reduced both amyloid and tau pathology, while single-cell RNA sequencing (RNA-seq) identified disease-associated MHC-II-expressing microglial populations selectively enriched in chimeras harboring *APOE4* neurons, highlighting a coordinated neuron-microglia interaction that promotes disease progression [20]. In a complementary study, introduction of the protective *APOE-*R136S (Christchurch) variant into the *APOE4* background rescued tau pathology, neurodegeneration, and neuroinflammation, while identifying heparan sulfate proteoglycan (HSPG)-mediated tau uptake as a key pathogenic mechanism and potential therapeutic target [21].

This ability to examine genetically defined human neurons within an *in vivo* brain environment has also uncovered mechanisms associated with other genetic forms of AD. Neurons carrying the familial AD mutation *APP* V717I exhibited altered transcriptional programs and allowed identifying LINGO2 as a regulator of synaptic dysfunction. Knockdown of *LINGO2* in transplanted neurons restored neurite morphology and reversed dysregulation of multiple AD-associated pathways, including apoptosis, cellular senescence, and ERK/MAPK signaling [22]. Likewise, transplantation of neurons derived from individuals with Down syndrome, a condition characterized by constitutive *APP* triplication, revealed impaired dendritic spine pruning and reduced synaptic stability compared with control neurons, demonstrating that intrinsic neurodevelopmental abnormalities persist following integration into the adult brain [23]. Overall, these studies show how chimeric models can separate cell-intrinsic effects of human AD-associated variants from those imposed by the surrounding brain environment, while simultaneously capturing interactions with host cells.

Importantly, mechanistic insights obtained from neuronal chimeras can also enable *in vivo* validation of disease pathways as potential therapeutic targets. Pharmacological inhibition of necroptosis prevented *MEG3*-driven neuronal death in AD chimeric mice [12], providing support for RIPK1 as a potential therapeutic target. Notably, CNS-penetrant RIPK1 inhibitors have subsequently entered clinical development, including DNL747, which reached early-stage clinical evaluation in AD (NCT03757325), Likewise, *LINGO2* downregulation rescued neuronal dysfunction in *APP* V717I chimeras [22], while engineering the protective *APOE*-R136S variant identified HSPG-mediated tau uptake as a therapeutically tractable pathway [21].

Together, neuronal chimeras provide a means to identify human-specific neuronal vulnerabilities, determine how genetic risk modifies these responses, and test the causal contribution of candidate pathways within the multicellular environment of the living brain.

### Microglia AD chimeras

Microglia are the brain's resident immune cells and central mediators of neuroinflammation in AD. Early studies demonstrated that hPSC-derived microglia progenitors engraft the mouse brain and progressively acquire transcriptional identities resembling primary human microglia [24, 25], laying the foundation for human microglia AD chimeras [26–29]. Efficient engraftment requires cytokine-humanized strains such as MITRG mice expressing human M-CSF, IL-3/GM-CSF, and thrombopoietin, or human CSF1 knock-in Rag2 knockout mice, frequently crossed with amyloid models such as transgenic APP/PS1 or *App* NL-G-F knock-in backgrounds [26, 28]. More recently, microglia-deficient *Csf1r* ΔFIRE/ΔFIRE mice have enabled near-complete replacement of endogenous microglia without the need for pharmacological depletion, providing an improved platform for studying human microglial biology *in vivo* [29, 30].

Human microglial chimeras have been particularly informative in distinguishing conserved from human-specific neuroimmune responses to AD pathology. Transcriptomic analyses of these chimeras revealed that plaque-associated human microglia adopt activation states that partially overlap with disease-associated microglia (DAM) described in mouse models while also displaying distinct human-specific transcriptional signatures. These states include gene programs related to antigen presentation, interferon and cytokine signaling, lipid metabolism, and phagocytic function, with *TREM2* signaling and *APOE* genotype emerging as major regulators of these activation states [26–28, 31]. Thus, rather than simply recapitulating murine DAM responses, human microglia adopt partially overlapping but molecularly distinct states within the amyloid-containing brain, revealing species-specific features of the neuroimmune response.

Importantly, these models also move beyond transcriptional comparisons by enabling the causal contribution of human microglia to AD pathology to be tested *in vivo*. In microglia-deficient amyloid models, the absence of microglia alters plaque organization and reduces the formation of compact plaque cores. Reconstitution of these brains with transplanted human microglia restores plaque-associated microglia clustering and remodels plaque architecture [29, 30]. Likewise, transplantation of microglia carrying AD-risk variants including *TREM2* mutations and *APOE4* modifies plaque morphology and exacerbates neuritic dystrophy [26, 32]. Together, these findings demonstrate that human microglia can actively modify amyloid organization and its associated neuronal damage, while providing an *in vivo* framework to determine how human AD-risk variants alter these functions.

The multicellular *in vivo* environment further enables to examine the dynamic microglial response to interacting AD pathologies. Combined amyloid–tau PS19 chimeric models have revealed that amyloid and tau pathology initially elicit distinct microglia activation programs that progressively converge as neurodegeneration advances [29]. Conversely, experimental depletion of microglia in amyloid models can redirect amyloid deposition toward vascular compartments and aggravates associated pathologies, supporting a context-dependent protective role for microglia during disease progression [33].

Human microglial chimeras are also beginning to provide platforms for evaluating how human microglia respond to disease-modifying therapies and for testing engineered microglia as cell-based interventions. In *App* NL-G-F *Csf1r* ΔFIRE/ΔFIRE chimeric mice reconstituted with human microglia, treatment with lecanemab induced robust phagocytic and lysosomal activation programs, resulting in reduced Aβ burden and attenuation of neuritic dystrophy, demonstrating that transplanted human microglia actively contribute to antibody-mediated amyloid clearance [34]. In a distinct therapeutic approach, CRISPR-engineered microglia-like cells expressing the Aβ-degrading enzyme neprilysin under the control of the plaque-responsive *CD9* promoter reduced Aβ burden, astrogliosis, neuroinflammation, and plasma neurofilament light chain levels, while preserving synaptic integrity. Combining neprilysin delivery with a *CSF1R* inhibitor-resistance approach enabled widespread engraftment throughout the brain and further enhanced therapeutic efficacy by reducing plaque burden and neuronal loss [35].

Collectively, human microglial chimeras reveal both conserved and human-specific neuroimmune responses to AD pathology and enable the causal contribution of human microglia and AD-associated genetic variants to disease progression to be examined *in vivo*. Importantly, their applications now extend beyond mechanistic disease modeling. They can be used to determine how human microglia participate in the response to established therapies, as illustrated by lecanemab, or, in a distinct xenotransplantation paradigm, to evaluate genetically engineered microglia as therapeutic agents capable of sensing pathology and delivering targeted interventions.

### Astrocyte AD chimeras

Astrocytes undergo profound morphological, molecular, and functional alterations throughout AD. Their normally homeostatic properties are progressively replaced by reactive phenotypes characterized by hypertrophy, increased expression of inflammatory mediators, and impaired neuroprotective functions. Compared with rodents, human astrocytes exhibit greater morphological complexity, larger territorial domains, and distinct transcriptional programs, suggesting that they may contribute to disease pathogenesis in species-specific ways [36, 37], thereby highlighting the need for human cell-based models to investigate astrocyte biology in AD.

Human astrocyte chimeras have been generated by differentiating hiPSCs into human astrocyte progenitor cells (hAPCs) [38] and transplanting them into the frontal cortex of neonatal immunodeficient AD mice, typically APP/PS1 mice or *App* NL-G-F crossed with NOD-SCID or Rag2 knockout mice. Following engraftment, these transplanted cells integrate within the host brain and progressively mature, developing complex morphologies characterized by enlarged territories, elaborated processes, and vascular endfeet contacting blood vessels, thereby acquiring characteristic features of human astrocytes *in vivo*. Engrafted astrocytes also establish gap-junction connections with neighboring host cells and display electrophysiological properties consistent with functional astrocytes [39]. Interestingly, exposure of transplanted astrocytes to amyloid pathology revealed heterogeneous morphological responses similar to those observed in AD patient brains, including hypertrophic astrocytes with thickened processes and atrophic astrocytes displaying reduced structural complexity [39]. These findings provided the first evidence that human astrocytes can mount disease-relevant responses within the pathological environment of the AD brain.

More recent work has extended this approach from describing human astrocyte responses to establishing their causal contribution to AD pathology and determining how this is modified by genetic risk. Cruz-Sese and colleagues [40] transplanted isogenic hAPCs carrying *APOE3* or *APOE4* variants into neonatal immunodeficient AD mice. Five to six months after transplantation, transplanted cells differentiated into mature astrocytes that integrated extensively into the upper layers of the cortex. *APOE3* and *APOE4* astrocytes differed in APOE expression, and differentially affected AD progression. While *APOE3* astrocytes ameliorated Aβ burden, tau pathology, and neuritic dystrophy, *APOE4* astrocytes exacerbated these processes. Moreover, the two isoforms influenced microglial responses to Aβ pathology. *APOE4* astrocytes enhanced microglial clustering around Aβ plaques and promoted a DAM-like state, whereas isogenic *APOE3* astrocytes reduced clustering and supported a more homeostatic profile [40]. The use of isogenic human astrocytes within the same *in vivo* pathological context provides a particularly informative example of the mechanistic value of chimeric models, allowing genotype-dependent, cell-intrinsic effects to be identified. Importantly, these effects extend beyond astrocytes themselves: human astrocyte *APOE* genotype modifies amyloid and tau pathology, neuritic damage, and the response of host microglia, illustrating how human genetic risk can influence multicellular disease processes across species within the same brain. Although the protective effects associated with *APOE3* astrocytes raise the possibility that astrocyte states or *APOE*-associated pathways could be therapeutically manipulated, these experiments were designed primarily to interrogate disease mechanisms rather than to evaluate astrocyte replacement as a therapy. The use of engineered astrocytes as a cell-based intervention therefore remains a distinct and currently unexplored therapeutic strategy.

Together, neuronal, microglial, and astrocyte chimeras demonstrate that several cellular responses to AD pathology are species-specific and genetically modifiable. A major strength of these models is their ability to expose human cells to a complex pathological environment while preserving human-specific cellular properties. They enable human cell-intrinsic vulnerabilities and genotype-dependent responses to be examined together with their consequences for surrounding cells, providing mechanistic insights into the multicellular nature of AD. Moreover, these models can support therapeutic development through *in vivo* validation of human-relevant targets and responses to disease-modifying therapies, while emerging xenotransplantation approaches using engineered human cells offer additional opportunities for cell-based interventions.

## Chimeric models of Parkinson’s disease

PD is the second most common neurodegenerative disorder worldwide. Degeneration of dopaminergic neurons in the substantia nigra pars compacta (SNpc) and the consequent depletion of dopamine in the striatum lead to the classical motor symptoms of the disease: tremor, rigidity, and bradykinesia [41]. The main histopathological hallmark is the presence of intracellular inclusions known as Lewy bodies, whose major component is misfolded ɑ-synuclein (ɑSyn) [42]. The etiology of PD involves a complex interplay among aging, environmental factors, and genetic predisposition. Although most PD cases are sporadic, 5–10% are linked to genetic mutations in genes such as *SNCA* (encoding ɑSyn), *LRRK2*, *VPS35*, *PRKN*, *PINK1*, and *DJ-1* [43].

To model PD in animals, researchers have used neurotoxin-based and genetic-based approaches [44]. Classical models use neurotoxins such as 6-hydroxydopamine (6-OHDA) and 1-methyl-4-phenyl-1,2,3,6-tetrahydropyridine (MPTP) to rapidly induce the degeneration of the nigrostriatal pathway and consequent motor impairments [45]. Genetic and ɑSyn-based models include transgenic models carrying PD-associated mutations (e.g., *SNCA*, *LRRK2*), viral vector-mediated overexpression of wild-type or mutant human ɑSyn, and the intracerebral administration of ɑSyn oligomers or preformed fibrils (PFFs), which induce progressive αSyn aggregation and spreading [46].

Building upon these established models, chimeric models of PD provide a complementary approach by allowing human dopaminergic neurons and glial cells to be studied within the brain environment. These models can reveal human-specific mechanisms driving dopaminergic neuron degeneration and neuroimmune interactions, dissect functional consequences of PD-associated genetic variants, and assess the long-term survival, integration, and therapeutic efficacy of transplanted cells*.*

### Neuron PD chimeras

Human neuronal transplantation in PD has developed along two related but distinct applications: neuronal chimeras designed primarily to investigate human-specific and cell-autonomous mechanisms of PD, and a much larger body of xenotransplantation research focused on evaluating dopaminergic cell replacement.

In mechanistic studies, transplantation of hiPSC-derived midbrain dopaminergic (mDA) progenitors from PD patients provides an opportunity to determine whether disease-associated phenotypes persist or emerge when human neurons mature within the living brain. hiPSC-derived progenitors from both sporadic and familial PD patients successfully engraft and survive following transplantation [47–52]. However, neurons carrying pathogenic mutations such as *SNCA* A53T, *SNCA* triplication, or *LRRK2* G2019S progressively develop elevated αSyn levels, phosphorylated αSyn inclusions, impaired neuronal maturation, and reduced generation of mature tyrosine hydroxylase-positive (TH⁺) neurons [13, 14, 53]. In contrast, neurons derived from sporadic PD patients generally fail to develop overt αSyn pathology following transplantation [47], suggesting that the strong cell-autonomous phenotypes associated with some familial mutations are not readily reproduced by sporadic patient-derived cells under these experimental conditions. These studies illustrate a specific mechanistic contribution of neuronal chimeras: disease-associated human genetic backgrounds can be examined after prolonged maturation within a shared *in vivo* environment, allowing persistent cell-intrinsic phenotypes to be distinguished from those requiring additional aging- or disease-associated environmental factors. The emergence of αSyn pathology in genetically susceptible human neurons also has implications for autologous transplantation in familial PD, as pathogenic features retained by donor cells could potentially compromise their long-term therapeutic performance.

A distinct and considerably larger body of transplantation research has used human dopaminergic cells primarily as a therapeutic intervention. This approach originated more than four decades ago with transplantation of fetal ventral mesencephalic cells into rodent models of PD [54, 55], providing early evidence that dopaminergic cell replacement could restore motor function. Subsequent studies using hESC-derived mDA neurons identified donor cell specification and functional integration as major determinants of successful dopaminergic replacement [56–58]. Transplantation of poorly specified or undifferentiated hNPCs resulted in limited dopaminergic differentiation and increased the risk of tumor formation or the generation of unwanted neuronal populations, including serotonergic neurons associated with graft-induced dyskinesias [52, 59–61].

These early xenotransplantation studies with fetal tissue and hESCs, which have been extensively reviewed [62], established the basis for current approaches employing hiPSC-derived mDA progenitors. Given the critical need to generate lineage-restricted mDA progenitors prior to transplantation, considerable effort has focused on optimizing hiPSC developmental patterning and cell selection to enhance graft purity, dopaminergic differentiation, and safety [63–70]. Following transplantation into toxin-induced PD models, appropriately specified hiPSC-derived mDA progenitors survive, differentiate into mature TH⁺ neurons, integrate into host circuitry, and improve motor function in mice and rats across multiple behavioral paradigms [14, 47, 49–51, 53, 63–67, 69–76]. Importantly, these studies demonstrate that graft survival alone is insufficient for robust functional recovery; therapeutic benefit depends on appropriate neuronal maturation and extensive integration with host circuitry. Long-term survival, neuronal maturation, and functional recovery have also been demonstrated in MPTP-lesioned non-human primates [48, 72, 77, 78], providing an important translational step toward clinical application.

The anatomical location of the graft represents an additional determinant of circuit reconstruction. Although ectopic transplantation into the striatum remains the predominant strategy, recent studies have attempted to reconstruct the nigrostriatal pathway by engrafting mDA progenitors directly into the SNpc. In these studies, transplanted cells matured into electrophysiologically functional dopaminergic neurons and extended TH⁺ axons toward the striatum, accompanied by improvements in motor deficits in 6-OHDA-lesioned animals [72, 79, 80]. These approaches aim to restore the anatomical organization of the nigrostriatal pathway rather than simply provide local dopamine release within the striatum, although whether this results in superior long-term functional outcomes remains to be established. More recently, transplantation of hiPSC-derived midbrain organoids has provided an alternative strategy in which grafted tissue can survive, generate functional TH⁺ neurons, extend projections into the host brain, and ameliorate motor deficits in PD mice [81].

Thus, neuronal transplantation in PD illustrates both the value and the importance of distinguishing mechanistic chimeric models from therapeutic xenotransplantation. Patient-derived chimeras provide an *in vivo* platform for examining cell-intrinsic human disease phenotypes and the consequences of PD-associated genetic variants, whereas therapeutic xenotransplantation studies test whether appropriately specified human dopaminergic cells can survive, integrate, reconstruct dopaminergic circuitry, and restore function. The latter have provided the preclinical foundation for clinical evaluation of hPSC-derived mDA progenitor transplantation in PD.

### Microglia PD chimeras

Recent studies have established microglia PD chimeras by transplanting hiPSC-derived hematopoietic progenitors, myeloid precursors, or microglia into the mouse brain [82–84]. To date, these chimeras have primarily relied on αSyn-based models, either by directly injecting human αSyn oligomers [82] or PFFs [83], or by transplanting hiPSC-derived cells harboring genetic PD mutations [84]. By combining human microglia with defined forms of human αSyn pathology or PD-associated genetic variants within the living brain, these models provide an opportunity to dissect human-specific neuroimmune responses *in vivo*.

Following transplantation into adult or neonatal immunodeficient mice, hiPSC-derived hematopoietic progenitors and myeloid precursors successfully engraft and differentiate into microglia with morphological and transcriptional features characteristic of mature human microglia [82–84]. Importantly, this approach enables cell-intrinsic effects of PD-associated mutations to be examined independently of engraftment efficiency or maturation. The A53T-αSyn mutation does not impair engraftment or differentiation of myeloid precursors, with no major differences in cell density, distribution, morphology, αSyn expression, or expression of key mature microglial genes. Instead, A53T-αSyn selectively alters microglial state, promoting a pro-inflammatory profile characterized by increased expression of genes involved in oxidative stress, inflammatory activation, and senescence, together with reduced expression of genes associated with immunomodulation and cell division [84]. These findings indicate that a PD-associated αSyn mutation can intrinsically reprogram human microglial responses without compromising their capacity to populate and mature within the brain.

Chimeric models have also revealed that the functional consequences of human microglial activation depend strongly on the molecular form of αSyn to which they are exposed. Soluble αSyn oligomers induce inflammasome activation and a robust pro-inflammatory response associated with enhanced neuronal degeneration [82], whereas fibrillar αSyn elicits a markedly distinct response. In PFF-based models, transplanted human microglia limit the propagation of pathogenic αSyn along the nigrostriatal pathway, supporting a neuroprotective role during disease progression [83]. These findings indicate that human microglial responses are highly context-dependent, with distinct αSyn conformers eliciting functionally divergent neuroinflammatory and protective programs.

Importantly, these chimeras can also be used to investigate how human microglia respond to candidate disease-modifying therapies, without the transplanted cells themselves constituting the therapeutic intervention. Immune complexes formed by αSyn and anti-αSyn antibodies enhanced inflammasome activation in transplanted human microglia [82], revealing a potential inflammatory consequence of antibody-mediated targeting of pathological αSyn. Thus, human microglial PD chimeras provide a mechanistic platform for defining human-specific neuroimmune responses to αSyn pathology and genetic risk, while also enabling evaluation of the consequences of therapeutic modulation within the living brain.

### Clinical translation in PD

The robust preclinical evidence supporting the safety and efficacy of hiPSC-derived mDA progenitor transplantation has provided the foundation for the first clinical trials of hiPSC-based replacement therapies in PD. The pioneering Kyoto phase I/II study (jRCT2090220384), initiated in 2018, recently reported the first clinical outcomes following transplantation of allogeneic hiPSC-derived CORIN⁺-sorted mDA progenitors into the putamen of seven patients with PD [85, 86]. After two years of follow-up, the transplanted cells survived and produced dopamine, as evidenced by increased [^18^F]DOPA PET uptake in the transplanted putamen, with no serious adverse effects or graft overgrowth. Among the six patients included in the efficacy analysis, improvements in motor scores were observed in four patients in the OFF state and five in the ON state, providing initial evidence of potential clinical benefit [85].

Building upon these encouraging results, several additional clinical trials are currently underway (NCT06482268, NCT06422208, NCT06687837, NCT06344026, NCT06145711, NCT05901818), evaluating different cell manufacturing protocols, graft composition, transplantation strategies, immunosupression, and patient populations. Long-term follow-up will be required to establish graft durability, sustained functional benefit, safety, and the extent to which transplanted neurons remain resistant to the host disease environment. Thus, PD provides a particularly advanced example of how chimeric preclinical research can successfully drive the translation of regenerative medicine into clinical practice.

## Chimeric models of Huntington´s disease

HD is an autosomal dominant neurodegenerative disorder characterized by progressive motor, cognitive, and psychiatric impairment. It is caused by an expanded CAG repeat in the huntingtin (*HTT*) gene, which generates a mutant protein (mHTT) with a toxic gain of function. mHTT disrupts cellular homeostasis and ultimately leads to selective degeneration of striatal medium spiny neurons (MSNs) involved in motor control [87, 88].

A variety of experimental models have been developed to investigate HD pathogenesis and evaluate therapeutic strategies. Widely used mouse models include transgenic mice such as R6/2 and YAC128 mice, which express human mHTT, as well as knock-in models such as Q140 and zQ175 mice, in which an expanded CAG repeat is inserted into the endogenous gene [89, 90]. Although these models differ in disease severity and progression, they recapitulate key behavioral and molecular features of HD [89]. R6/2 mice display a rapid progressive neurological phenotype with severe motor deficits [91]. YAC128 mice show a more gradual course of neurodegeneration [92], while knock-in models exhibit a more progressive pathology that reflects the temporal dynamics of human HD [93, 94]. In addition, the quinolinic acid (QA)-lesioned rodent model is also used for preclinical evaluation of cell replacement strategies. Although this model does not replicate disease progression, it exhibits acute and rapid neurodegeneration of MSNs [95].

In addition, chimeric models of HD have predominantly focused on regenerative applications. Most studies have evaluated the capacity of hNPCs or striatal progenitors to survive, differentiate, integrate into host circuitry, and replace vulnerable neuronal populations. Thus, the HD literature discussed below largely represents therapeutic xenotransplantation, although selected studies have also provided insights into the cell-autonomous and non-cell-autonomous effects of mHTT in human neural cells.

### Neuron HD chimeras

Neurodegeneration in the striatum is the primary pathological hallmark of HD. Consequently, most studies focus on the transplantation of hNPCs and neurons into the striatum of murine HD models as a potential therapeutic strategy. Neuronal HD chimeras have been generated using hNPCs derived from hESCs and hiPSCs [96–100]. In most studies, hNPCs have been differentiated through protocols based on dual SMAD inhibition [98–105], achieving efficient neural conversion [106]. Others have also produced three-dimensional structures such as embryoid bodies (EBs) [98, 99, 102, 107], neurospheres [96, 97, 102] or even performed 3D differentiation using hydrogels [101]. Transplantation approaches also differ in the degree of regional specification achieved prior to grafting. While some studies differentiate hNPCs in a general manner, [96, 97, 99, 100, 107, 108], others pre-commit cells toward a MSNs fate before transplantation, thereby increasing the specificity of the injected cells [98, 101–105]. To this end, CD200-based cell sorting has been used to obtain a homogeneous population of transplantable striatal neuroblasts [103]. hNPCs are typically transplanted into the striatum, with timing varying across models. In aggressive models such as R6/2, cells are transplanted at early symptomatic stages [101, 105, 108], whereas in slower-progressing models, cell delivery occurs later [96, 97, 99, 107, 108]. Finally, QA-lesioned rodents typically receive intrastriatal transplantation of hNPCs after lesion induction [98, 100, 102, 104].

Across different experimental models and differentiation strategies, a consistent finding is that appropriately specified hNPCs can survive long term, mature within the striatum, and functionally integrate into host circuitry. Transplanted cells generate DARPP-32-positive neurons with transcriptional and electrophysiological features of MSNs and establish synaptic connections with host neurons. More recent studies further indicate that grafted human cells can generate distinct MSN subtypes and extend axonal projections toward cortical and other basal ganglia regions, supporting partial reconstruction of corticostriatal and striatal output circuitry [98, 102, 104, 107, 108]. Importantly, these structural and functional properties are accompanied by improvements in motor and behavioral phenotypes across multiple HD models [96–98, 100–102, 104, 107, 108]. Together, these studies suggest that successful cell replacement depends not simply on graft survival but on appropriate striatal specification, neuronal maturation, and integration into the distributed circuitry disrupted in HD.

Therapeutic benefit may also extend beyond direct neuronal replacement. Transplantation has been associated with increased BDNF signaling, reduced mHTT accumulation, and modulation of inflammatory responses [96, 98, 100, 108], suggesting that grafted cells can modify the host disease environment in addition to replacing lost neurons. Genetic engineering provides a further opportunity to enhance these effects. Editing *SUPT4H1*, a transcription factor involved in regulating CAG repeat expression, reduced mHTT levels without affecting wild-type HTT and improved motor function following transplantation of patient-derived hNPCs into YAC128 mice [99]. Similarly, hNPCs engineered to overexpress BDNF ameliorated HD-associated phenotypes following transplantation in mouse chimeras [105]. These approaches illustrate how therapeutic xenotransplantation can combine neuronal replacement with targeted modification of disease-associated pathways, although their long-term safety, stability, and comparative advantage over unmodified donor cells remain to be established.

### Glia HD chimeras

HD has traditionally been studied as a neuronal disorder, but accumulating evidence indicates that glial dysfunction also contributes to disease pathogenesis. Alterations in white matter and myelination occur early in HD, and transcriptomic analyses of patient brains have revealed substantial changes in glial gene-expression programs [109–112]. Glial cells express mHTT and display functional impairments affecting homeostatic support, myelination, and neuroinflammatory responses that may exacerbate neuronal vulnerability [113–117]. In this context, humanized chimeric models incorporating human glial cells provide a powerful platform to elucidate their role in HD and assess their potential for cell-based therapies.

To date, only a few studies have generated glial HD chimeras, primarily through transplantation of human glial progenitor cells (hGPCs), whereas direct transplantation of mature astrocytes, microglia or oligodendrocytes has not yet been reported. Control or mHTT-expressing hGPCs have been engrafted into wild-type, R6/2, or hypomyelinated host mice, allowing their intrinsic developmental properties and their interactions with diseased or healthy host environments to be examined [15, 118–120]. Most studies have used neonatal transplantation to achieve extensive glial colonization, targeting either white matter regions to investigate myelination or the striatum to examine glia-neuron interactions. These chimeras have provided direct evidence that mHTT induces cell-intrinsic abnormalities in hGPCs *in vivo*. In hypomyelinated mice, mHTT-expressing hGPCs exhibited impaired glial differentiation, with deficient astrocyte development and myelination [15]. Importantly, because mutant and control human cells were examined within comparable host environments, these experiments could attribute the observed developmental and myelination defects directly to mHTT expression in human glia.

Conversely, transplantation of healthy hGPCs has revealed that modifying the glial environment can influence neuronal dysfunction and disease progression. Healthy hGPCs transplanted into R6/2 mice progressively colonized the striatum and partially replaced resident glial populations, improving motor and cognitive performance, extending survival, and enhancing the structural complexity and function of host MSNs [118, 120]. These findings provide evidence for a non-cell-autonomous contribution of glia to HD pathology: replacing dysfunctional glial support with healthy human glia can modify the phenotype of genetically diseased host neurons.

More recent work has extended this concept from disease modification to competitive glial replacement. Vieira and colleagues generated chimeric mice containing human glia established by neonatal transplantation and subsequently introduced young healthy hGPCs into the adult striatum. The newly transplanted cells progressively outcompeted and replaced both healthy and mHTT-expressing human glia, indicating that competitive fitness is strongly influenced by cellular age rather than disease genotype alone [119]. This ability of young hGPCs to replace pre-existing adult human glia suggests a potential strategy for therapeutic glial replacement, while also revealing fundamental principles governing competition between human glial populations *in vivo*.

Collectively, HD glial chimeras illustrate particularly well the dual value of humanized models. Mechanistically, they demonstrate that mHTT produces cell-intrinsic defects in human glial differentiation and myelination and that glial dysfunction can non-cell-autonomously influence neuronal pathology. Therapeutically, they show that healthy hGPCs can modify the diseased neural environment and, under appropriate conditions, competitively replace pre-existing glial populations.

## Chimeric models of Amyotrophic Lateral Sclerosis

ALS is a progressive and fatal neurodegenerative disorder characterized by the selective degeneration of motor neurons (MNs) in the cerebral cortex, brainstem and spinal cord [121–124]. This loss is accompanied by neuroinflammation and a complex interconnected web of cellular dysfunctions, including intracellular protein aggregation, oxidative stress, mitochondrial damage, and glutamate-mediated excitotoxicity [121, 123, 125]. Approximately 90–95% of ALS cases are classified as sporadic (sALS), whereas the remaining 5–10% represent familial ALS (fALS), with multiple disease associated genes identified to date [126]. The most common genetic cause, responsible for roughly 40% of familial and 7% of sporadic cases, is a GGGGCC hexanucleotide repeat expansion (HRE) within the non-coding region of the *C9ORF72* gene [124, 127, 128]. Mutations in *SOD1* account for approximately 20% of fALS cases, while other major ALS-associated genes include *TARDBP* (encoding TDP-43), *FUS* and *TBK1*, alongside less frequent mutations in *VCP*, *SQSTM1* and *OPTN* [124].

Despite major advances in understanding ALS, its etiopathogenesis remains incompletely understood and available treatments provide limited therapeutic benefit. Rodent models carrying *TDP-43, FUS,* and *C9ORF72* mutations are increasingly used to study ALS, while transgenic mice and rats overexpressing mutant *SOD1*, particularly the widely characterized SOD1-G93A model, remain widely used for investigating disease mechanisms and evaluating preclinical therapeutics [129, 130]. Humanized ALS chimeras have also been developed to investigate human-specific and cell-autonomous disease mechanisms, as well as interactions between human neurons or glia and the host environment. In parallel, a distinct body of xenotransplantation studies has evaluated human neural and glial cell populations primarily for their potential neuroprotective or regenerative effects.

### Neuron ALS chimeras

In contrast to AD and PD, relatively few studies have used transplanted human neurons to investigate human-specific mechanisms of ALS *in vivo*. Instead, transplantation of hNPCs into ALS models has primarily been developed as a therapeutic strategy, with transplanted cells evaluated for their capacity to survive and integrate within the diseased spinal cord and to provide neuroprotective effects. Early studies established that hNPCs can survive, migrate, and differentiate following transplantation into SOD1-G93A models. Dual cervical and lumbar transplantation of hNPCs into presymptomatic SOD1-G93A rats delayed motor symptoms and prolonged survival [131], while hNPCs transplanted into the lumbar spinal cord survived and differentiated into mature neurons in both wild-type and ALS rats [132]. These studies demonstrated that the ALS spinal cord can support long-term survival and neuronal differentiation of transplanted hNPCs. Consistent with previous findings, transplantation of aldehyde dehydrogenase-enriched hNPCs into SOD1-G93A mice improved motor unit function and prolonged survival following either intrathecal or systemic administration. These effects were associated with increased neurotrophic support and reduced gliosis and astrocyte activation [133]. Together, these findings suggest that the therapeutic effects of transplanted hNPCs in ALS models may arise largely from modulation of the host microenvironment rather than direct replacement of degenerating motor neurons.

### Astrocyte ALS chimeras

Despite the selective loss of MNs in ALS, there is growing evidence that dysfunctional astrocytes contribute to disease progression through non-cell-autonomous mechanisms. Chen and colleagues differentiated hESCs and hiPSCs from healthy individuals and ALS patients into hNPCs and transplanted them into the adult mouse spinal cord. The transplanted cells proliferated, migrated extensively, and differentiated predominantly into astrocytes that structurally and functionally integrated into the host tissue, progressively replacing a substantial proportion of resident mouse astrocytes over nine months. Notably, mice receiving cells derived from ALS patients developed motor deficits, whereas those transplanted with cells from healthy individuals did not show comparable locomotor impairment [134]. By placing control and ALS-derived human astrocytes within a comparable *in vivo* environment, this study provided direct evidence that disease-associated properties intrinsic to human ALS astrocytes can contribute to motor dysfunction. More broadly, it illustrates how chimeric models can establish the causal contribution of human non-neuronal cells to a disease defined by selective neuronal degeneration.

Other studies have used glial transplantation as a therapeutic intervention. Astrocyte and glial progenitor transplantation has produced variable outcomes in preclinical ALS models. Lepore and colleagues found that rat glial precursors restored GLT1-mediated glutamate transport, attenuated MN loss and prolonged survival, whereas human fetal glial precursors transplanted using a related strategy showed no comparable functional benefit [135, 136]. Differences in host species, immunosuppression, graft dose and injection strategy may have contributed to these divergent outcomes. Subsequent studies using hESC-derived hAPCs or hiPSC-derived glial-rich neural progenitors reported delayed disease progression and improved motor performance, with protection attributed to glutamate clearance, neurotrophic support, modulation of oxidative stress and gliosis [137, 138]. These findings suggest that the potential therapeutic benefit of glial transplantation may derive primarily from neuroprotection and modification of the host microenvironment rather than replacement of the degenerating MN populations.

Interestingly, an engineered approach using GDNF-secreting cortical hNPCs protected upper and lower MNs and extended survival in SOD1-G93A rats, while transplantation into the macaque motor cortex further demonstrated graft survival and GDNF production without overt adverse effects [139]. These findings support the feasibility of using engineered hNPCs for sustained local delivery of neuroprotective factors, while further studies are required to establish the durability and functional relevance of these effects.

Together, these studies illustrate the complementary but distinct uses of human glial transplantation in ALS. Disease-modeling chimeras can reveal the causal contribution of human astrocyte dysfunction to MN pathology, whereas therapeutic xenotransplantation evaluates whether healthy or engineered glial populations can modify the diseased environment and protect vulnerable neurons.

### Microglia ALS chimeras

Neuroinflammation and microglia are active players in ALS pathogenesis and progression, but the underlying mechanisms remain poorly understood. Recent human microglial chimeras have provided an opportunity to functionally interrogate disease-associated transcriptional states identified directly in ALS patient tissue. Masrori et al. performed single-nucleus RNA-seq of the motor cortex and spinal cord from healthy subjects and patients with sALS or *C9ORF72* ALS. They found that *C9ORF72* was highly expressed in microglia and that the HRE led to reduced *C9ORF72* expression levels. Moreover, sALS microglia transitioned toward disease-associated reactive states, whereas *C9ORF72* HRE microglia exhibited reduced activation and alterations in endolysosomal and phagocytic pathways [123]. Importantly, these patient observations were recapitulated and mechanistically investigated *in vivo* using human microglial chimeras. hiPSC-derived microglia carrying either *C9ORF72* loss-of-function or the C9ORF72 HRE, as well as isogenic controls, were transplanted into the mouse brain. Both genetic perturbations reproduced defects in microglial degradative pathways, supporting *C9ORF72* loss-of-function as a cell-intrinsic driver of impaired endolysosomal and phagocytic function in human microglia. Thus, the combination of patient transcriptomics, isogenic hiPSC models and humanized chimeras provides a particularly powerful framework for moving from associations in human tissue to mechanistic interrogations.

Interestingly, single-nucleus RNA-seq of patient tissue also revealed reduced activation states in astrocytes from *C9ORF72* HRE carriers compared with sALS and identified a specific astrocyte transcriptional state enriched in the spinal cord. These observations suggest that *C9ORF72*-associated dysfunction may extend beyond microglia and raise the possibility that region-specific astrocyte states contribute to the selective vulnerability of the spinal cord. However, these astrocyte-associated mechanisms have not yet been functionally tested in chimeric models and therefore remain an important question for future studies.

### Clinical translation in ALS

Preclinical xenotransplantation studies demonstrating the survival and neuroprotective potential of human neural and glial progenitors in ALS models have supported the clinical evaluation of several cell-based strategies. Early phase I/II studies evaluated fetal spinal cord-derived hNPCs delivered directly into the lumbar and cervical spinal cord of patients with ALS. These studies established the feasibility of intraspinal transplantation and reported no major graft-related safety concerns during long-term follow-up (NCT01348451; NCT01640067) [140–142].

More recent trials have translated glial and engineered-cell strategies evaluated in preclinical xenotransplantation studies described above. The phase I/IIa AstroRx® trial (NCT03482050) evaluated intrathecal administration of hESC-derived astrocytes and found the treatment to be generally safe and well tolerated, with a transient signal of reduced functional decline during the first three months after administration, supporting further evaluation [143]. In a complementary strategy, a phase I/IIa study (NCT02943850) transplanted hNPCs engineered to secrete GDNF into the lumbar spinal cord. The primary safety endpoint was met and post-mortem analyses demonstrated long-term graft survival and GDNF production for up to 42 months, although benign neuromas were observed near injection sites in some participants [144]. Together, these early clinical studies illustrate the translation of two distinct therapeutic concepts emerging from preclinical xenotransplantation: modification of the diseased glial environment through transplantation of healthy astrocytes and sustained local delivery of neuroprotective factors using engineered human cells. However, whether either approach can produce durable clinical benefit remains to be established.

Overall, ALS chimeric studies highlight the contribution of human glial dysfunction to disease pathogenesis, revealing cell-intrinsic and non-cell-autonomous mechanisms. In parallel, therapeutic xenotransplantation studies have established the feasibility of using healthy or engineered human neural and glial cells to modify the diseased CNS environment, with some of these approaches now progressing into early clinical evaluation.

## Chimeric models of Multiple Sclerosis and demyelinating diseases

MS is a chronic, multifactorial CNS disorder driven by a complex interplay of immune, genetic, and environmental factors, and is a leading cause of neurological disability in young adults. Characterized by immune-mediated demyelination, neuroinflammation, and neurodegeneration, MS pathology involves the activation of lymphocytes, macrophages, microglia, and astrocytes, ultimately disrupting axonal conduction and metabolic support [145–147]. Current disease-modifying therapies primarily target immune responses, but do not effectively promote remyelination or prevent neurodegeneration [145]. Although MS is the most prevalent demyelinating disorder, this group of diseases also includes other inflammatory conditions such as progressive multifocal leukoencephalopathy (PML) caused by John Cunningham (JC) virus infection of the CNS [148], as well as inherited leukodystrophies, characterized by impaired myelin maintenance and dysfunction of the axon-glia unit [149, 150]. Among these, Canavan disease (CD), adult-onset leukoencephalopathy with axonal spheroids and pigmented glia (ALSP), and metachromatic leukodystrophy (MLD), together with MS and PML, are currently being investigated using humanized chimeric models [151–157]. Despite their diverse etiologies, these disorders converge on dysfunction or loss of myelin and myeling-forming and supporting glial populations, making human glial chimeras particularly valuable for investigating human-specific mechanisms of myelin formation, injury, and repair, as well as for evaluating glial replacement and remyelination strategies.

A broad range of demyelinating mouse models have been used as hosts for human cell transplantation, with the choice of model determined by the biological or therapeutic question being addressed [158, 159]. Genetic hypomyelinating models such as the shiverer mouse, characterized by loss of myelin basic protein, provide a permissive environment to study the myelinating capacity of transplanted human cells [160]. Immunodeficient strains, including Rag2 knockout mice [161], are widely used as permissive hosts and can be combined with toxin-induced demyelination methods, such as the demyelinating toxin lysophosphatidylcholine/lysolecithin (LPC) [162–164], which generate focal myelin lesions, and cuprizone, which results in diffuse demyelination [165, 166]. In addition, the shiverer x Rag2 knockout mouse integrates hypomyelination with immune permissiveness, making it particularly suitable for cell transplantation studies [167]. In parallel, inflammatory demyelination has been modeled using the experimental autoimmune encephalomyelitis (EAE) model, induced by immunization with myelin antigens [168]. Infection-associated demyelination is represented by viral models, including infection with the JHM strain of mouse hepatitis virus (JHMV), [169], as well as JC virus, the causative agent of PML [153]. Genetic models of leukodystrophies, including ARSA-deficient mice for MLD, ASPA-deficient Nur7 mice for CD, and microglia-deficient *Csf1r* ΔFIRE/ΔFIRE mice displaying features relevant to ALSP, further enable disease-specific consequences of human cell engraftment to be investigated [151, 170–172]. Together, these complementary host environments allow human cells to be interrogated across distinct pathological contexts, from developmental hypomyelination to inflammatory, infectious, and genetic demyelination. Importantly, as in the other disorders discussed above, these studies encompass both mechanistic chimeric models designed to investigate human cell biology *in vivo* and therapeutic xenotransplantation paradigms aimed primarily at restoring myelin or replacing dysfunctional glial populations.

### Neural progenitor cell-based chimeras in demyelinating diseases

hNPC transplantation has been explored across demyelinating disorders primarily as a therapeutic strategy to promote CNS repair. hNPCs can act through multiple mechanisms including immunomodulation, neuroprotection, trophic support, cell replacement and, in some contexts, direct remyelination. The relative contribution of these mechanisms depends strongly on the pathological environment and remains incompletely defined.

Humanized models have been generated using hNPCs derived from hESCs, hiPSCs, and fetal hNPCs [173]. hNPCs have been commonly obtained through dual SMAD inhibition protocols [152, 174, 175], spontaneous neural differentiation without patterning factors [157], or 3D culture approaches such as EBs [176, 177] and neurospheres [173, 178]. Across studies, these cells are typically maintained as immature neural populations, without strict pre-specification toward defined subtypes. Transplantation strategies are heterogeneous and vary according to disease model. Importantly, injection sites extend beyond the brain to the spinal cord, reflecting the widespread nature of CNS white matter damage. In inflammatory models such as EAE and viral-induced demyelination, intraspinal transplantation is generally performed in young adult mice [174, 176, 177]. Nevertheless, despite the prominent involvement of the spinal cord in MS, chimeric approaches in this region have so far been used primarily to evaluate the reparative or immunomodulatory effects of transplanted cells, whereas their potential to dissect human-specific mechanisms of demyelination, remyelination failure and glia-immune interactions within the spinal cord remains comparatively underexplored. In genetic hypomyelination and leukodystrophy models, early postnatal transplantation is instead typically employed to maximize widespread CNS engraftment. In MLD and CD models, cells are commonly delivered via single intracerebroventricular [157] or multisite bilateral injections in different brain regions [152, 175]. Similarly, in shiverer mice, hNPCs were transplanted into multiple bilateral white matter regions in neonatal and juvenile animals [173].

A major insight emerging from these studies is that functional recovery does not necessarily require direct replacement of myelinating cells. Early studies in EAE and viral models showed that hNPC transplantation improves recovery primarily through immunomodulatory mechanisms including reduced neuroinflammation and recruitment of regulatory T cells, whose depletion diminished the beneficial effects of transplantation [174, 176–178]. Notably, the relationship between remyelination and functional recovery remains complex, as increased remyelination does not always translate into significant clinical benefits [176, 177]. In other pathological contexts, however, hNPCs can contribute directly to myelin repair. In shiverer and LPC-lesioned mice, transplanted cells differentiated into myelinating human oligodendrocytes (hOLs), generating graft-derived myelin and supporting long-term repair [173].

Genetic engineering further expands the therapeutic functions of transplanted hNPCs beyond cell replacement. In leukodystrophies, ARSA-overexpressing hNPCs reduced sulfatide accumulation following transplantation in the MLD model, effectively acting as long-term enzyme delivery systems [157]. Similarly, ASPA-expressing hNPCs improved myelination and motor function in the CD model, even when administered at symptomatic stages [152, 175]. These studies illustrate how transplanted hNPCs can serve not only as sources of replacement of cells but also as sustained vehicles for therapeutic protein delivery.

The translational potential of hNPC transplantation has also begun to be explored clinically. A Phase I study in four patients with Pelizaeus-Merzbacher disease, a rare congenital X-linked recessive leukodystrophy, reported that hNPC transplantation was well tolerated and was associated with MRI evidence of myelination and modest neurological improvement in three of the four participants [179].

Together, these studies position hNPC xenotransplantation as a versatile therapeutic platform in demyelinating disorders, with effects ranging from immunomodulation and direct myelin repair to sustained delivery of disease-correcting enzymes.

### Glial progenitor cell-based chimeras in demyelinating diseases

hGPC-based chimeric models are a valuable platform for assessing human glial contributions to myelin formation, remyelination, and repair *in vivo*, while also enabling the evaluation of glial replacement strategies. Unlike broader hNPC populations, hGPCs are intrinsically biased toward astrocytic and oligodendroglial lineages, making them particularly suitable for dissecting human glial behavior and reconstructing damaged white matter [180].

hGPCs have been primarily isolated from human fetal forebrain tissue using immunomagnetic sorting strategies [153, 156, 167, 181], while hGPCs derived from established cell lines have also been employed [182]. Transplantation strategies using hGPCs in shiverer mice have evolved from early developmental approaches aimed at widespread CNS engraftment to more targeted and functionally oriented delivery paradigms. In neonatal animals, cells are typically delivered using multifocal intracerebral or intraventricular injections [153, 167, 181, 182]. More recently, transplantation has been extended to young shiverer mice via bilateral corpus callosum injections, and to adult shiverer mice and wild-type mice with cuprizone-induced demyelination to assess remyelination in acquired white matter lesions [156].

A major insight from these chimeras is the remarkable capacity of hGPCs to colonize the host CNS and reconstruct white matter. In the shiverer model, neonatal hGPC transplantation led to extensive brain engraftment and formation of ultrastructurally normal myelin, improving survival and neurological function [167]. Subsequent work confirmed that hGPCs outcompeted and replaced the host GPCs, generating a human population able to differentiate into human oligodendrocytes (hOLs) [181]. Importantly, this capacity is not restricted to developmental hypomyelination. In both shiverer mice and cuprizone-lesioned adult mice, pre-engrafted hGPCs responded to injury by activating oligodendroglial programs and enhancing remyelination, indicating responsiveness to demyelinating cues [156]. Together, these findings demonstrate that hGPCs retain the ability to sense pathological cues and generate myelinating oligodendrocytes within both the developing and adult CNS.

These studies also indicate that functional benefit cannot be attributed exclusively to direct myelin replacement. In shiverer mice, hGPC transplantation improved survival and neurological function even in the absence of early mature myelin formation, suggesting additional reparative mechanisms beyond myelin production [182]. This uncoupling between structural remyelination and functional recovery highlights the importance of distinguishing direct cell replacement from broader effects of transplanted human glia on the host tissue.

Beyond regenerative applications, human glial chimeras have provided mechanistic insights into diseases that cannot be faithfully modeled in conventional rodents. In a humanized model of PML, intracerebral JC virus infection selectively targeted human astrocytes and hGPCs, supporting the idea that these cells represent primary viral targets, whereas demyelination occurs secondarily following oligodendroglial apoptosis [153].

The concept of glial humanization has more recently been extended to microglia-associated white matter disease. In a xenotolerant model of ALSP, transplantation of hiPSC-derived microglia progenitors into *Csf1r* ΔFIRE/ΔFIRE mice restored homeostatic microglial signatures and prevented white matter abnormalities. Notably, CRISPR-corrected ALSP patient-derived microglia progenitors ameliorated established pathology [151], providing a direct link between correction of a disease-associated human glial genotype and restoration of white matter homeostasis. These findings underscore the utility of hGPC-based chimeras for modelling human white matter biology, dissecting mechanisms of repair, and providing a framework for glial-based regenerative strategies.

### Oligodendrocyte progenitor cell-based chimeras in demyelinating diseases

Complementary strategies have focused on human oligodendrocyte precursor cells (hOPCs), a lifelong resident glial population that generates oligodendrocytes and supports myelination, remyelination and CNS plasticity [183, 184]. Their lineage commitment and intrinsic myelin-forming capacity make hOPCs an attractive therapeutic candidate for demyelinating diseases [185].

Despite their therapeutic promise, the generation of hOPCs from hESCs and hiPSCs remains technically challenging. For the generation of humanized models, hOPCs have traditionally been derived using classic developmental patterning protocols [152, 155, 186–192]. However, these approaches often yield heterogeneous and inefficient hOPC populations, limiting their reproducibility and scalability. hOPCs have also been generated using more recent transcription factor-driven strategies, enabling more rapid and efficient differentiation [154, 162, 193].

Transplantation approaches have been adapted to address different aspects of therapeutic remyelination. Early studies employed neonatal transplantation in shiverer mice and bilateral injections to maximize oligodendroglial engraftment [154, 155, 186, 187, 192, 193]. More extensive multisite transplantation paradigms have also been employed in neonatal CD model mice [152, 194]. Additional approaches have extended transplantation to juvenile or adult shiverer mice [162] and adult LPC models [155]. In inflammatory demyelination, hOPCs have been administered via intracerebroventricular injection during active EAE [188, 189]. Importantly, transplantation strategies have also been extended to non-human primates [190].

Across these models, a consistent finding is the robust intrinsic capacity of hOPCs to generate mature, myelinating oligodendrocytes *in vivo*. In neonatal and juvenile shiverer-based models, transplanted hOPCs extensively engrafted white matter tracts, differentiated along the oligodendroglial lineage, and generated ultrastructurally compact myelin, supporting functional restoration of hypomyelinated CNS tissue [154, 162, 186, 192, 193]. Similar results have been observed following hOPC transplantation into organotypic shiverer brain slice cultures, confirming their robust *ex vivo* myelination capacity [191, 195]. However, therapeutic outcome is strongly context dependent. Developmentally permissive hosts support extensive migration and myelination, whereas established adult lesions provide a more restrictive environment, indicating that the regenerative potential of donor cells depends not only on their intrinsic myelinogenic capacity but also on their ability to respond to local pathological cues.

Beyond remyelination, hOPCs also exert immunomodulatory effects in EAE models, reducing neuroinflammation and clinical severity despite limited myelin repair, suggesting additional host immune modulation [188, 189]. Together with similar observations from hNPC and hGPC transplantation, these findings reinforce the concept that structural remyelination and functional recovery are not necessarily equivalent outcomes and that transplanted human glial cells can modify the host environment through multiple mechanisms.

Recent studies have therefore focused on enhancing donor-cell performance. CRISPR-based approaches have been used to enhance hOPC migration and function in LPC models [155], suggesting that genetic modification can reprogram hOPC-lesion interactions. Similarly, ASPA-corrected hOPCs achieved widespread engraftment and improved myelination in leukodystrophy models [152, 194]. Moreover, CRISPR/Cas9-mediated HLA engineering has generated hypoimmunogenic hOPCs with reduced immune rejection [194], addressing one of the major barriers to the broader application of allogeneic cell replacement strategies. Beyond genetic engineering, metabolic modulation can influence hOPC maturation, with metformin-driven mitochondrial enhancement promoting maturation and highlighting metabolism as a regulator of remyelination efficiency [187]. Overall, these findings indicate that hOPCs are a promising source for remyelination therapies due to their intrinsic myelinogenic capacity, but their therapeutic performance depends on adaptation to the lesion microenvironment. Genetic, immunological, and metabolic modulation may therefore enhance the ability of transplanted hOPCs to function within adult or diseased CNS tissue.

## Current limitations and future directions

**Fig. 2:**
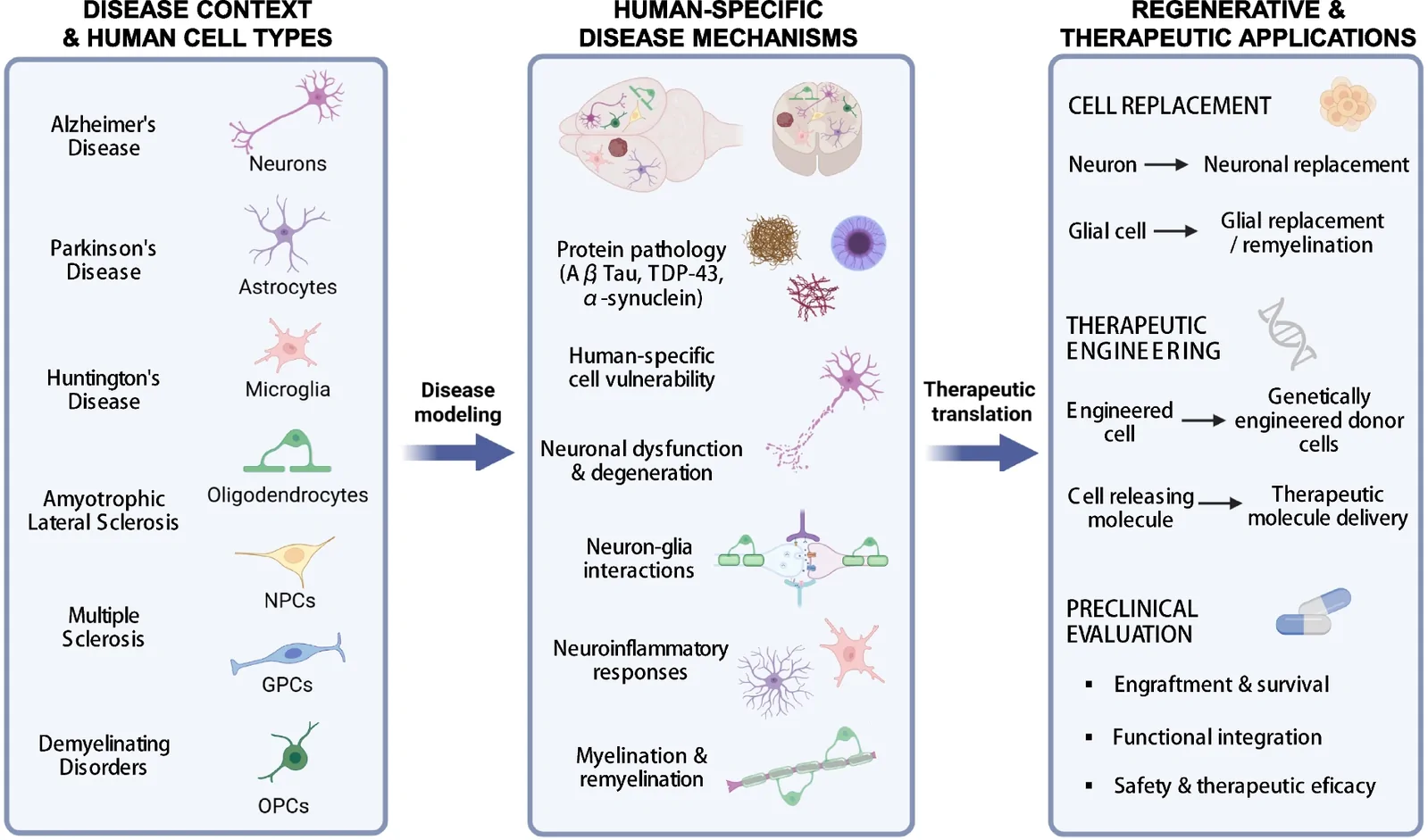
**Human CNS chimeric models bridge mechanistic discovery and regenerative medicine. **Human neural chimeric models provide complementary platforms for investigating human-specific mechanisms across neurodegenerative and demyelinating diseases, including Alzheimer’s disease, Parkinson’s disease, Huntington’s disease, Amyotrophic Lateral Sclerosis, Multiple Sclerosis, and other demyelinating disorders. Depending on the disease context and transplanted human cell population, these models enable the study of human-specific pathological features, including protein aggregation, selective cellular vulnerability, neuronal dysfunction and degeneration, altered neuron-glia interactions, neuroinflammatory responses, and defects in myelination and remyelination. Beyond disease modeling, xenotransplantation approaches provide platforms for evaluating regenerative and cell-based therapeutic strategies, including neuronal and glial replacement, genetically engineered donor cells, and preclinical assessment of cell engraftment, functional integration, safety, and therapeutic efficacy. *Created with BioRender*

Chimeric models have transformed the study of neurodegenerative and demyelinating diseases by revealing human-specific cellular phenotypes, uncovering mechanisms that cannot be faithfully recapitulated in conventional experimental systems, and providing complementary platforms for mechanistic investigation and evaluation of regenerative therapeutic strategies (Fig. 2). Nevertheless, several biological and technical limitations must be addressed before these models can fully realize their mechanistic and translational potential.

A major limitation remains the strong dependence of engraftment on the host microenvironment. While neonatal and developmentally permissive brains consistently support widespread migration, maturation, and functional integration of transplanted cells, adult and diseased tissues are considerably less permissive, limiting both engraftment efficiency and therapeutic efficacy. In addition, current differentiation protocols often generate heterogeneous cell populations with variable maturation states, reducing reproducibility across studies and complicating comparisons between experimental platforms.

The choice of host model also remains a critical consideration. Although existing rodent models reproduce important aspects of disease biology, none fully captures the progressive, multicellular, and systemic nature of human neurodegenerative disorders. Moreover, transplanted human cells remain embedded within a predominantly rodent environment, creating cross-species interactions involving ligands, receptors, extracellular matrix components, vascular signals, and differences in developmental timing that may influence human cell behavior. Appropriate controls, including isogenic donor lines and comparisons with corresponding host-cell responses, are therefore particularly important for identifying human-specific phenotypes. Furthermore, most chimeric studies rely on immunodeficient hosts, precluding investigation of adaptive immune responses that are increasingly recognized as important contributors to disease pathogenesis and therapeutic outcomes. This is particularly relevant for disorders such as MS, in which peripheral immune cells, including T cells, are central drivers of pathology, but also for neurodegenerative diseases in which interactions between the peripheral and central immune systems are increasingly recognized. Consequently, current chimeric models largely capture interactions among CNS-resident human cell populations while failing to reproduce the contribution of the human adaptive immune system and its crosstalk with human neural and glial cells. Incorporating human peripheral immune components into future chimeric platforms will therefore be important for more faithfully modeling the multicellular and systemic nature of human disease.

Addressing these limitations will require the development of next-generation chimeric platforms with improved biological fidelity and translational relevance. Advances in CRISPR/Cas9 genome editing, transcription factor-guided lineage specification, metabolic programming, and the generation of hypoimmunogenic donor cells are already enabling the production of more homogeneous cell populations with enhanced survival, accelerated maturation, and improved functional integration following transplantation [35, 99, 105, 139, 144, 155, 157, 194]. These approaches also enable donor cells to be engineered not only for improved engraftment but also to correct disease-associated mutations, resist hostile pathological environments, or deliver therapeutic molecules in a spatially controlled manner. At the same time, the field is moving beyond single-lineage transplantation toward increasingly sophisticated multi-lineage humanized brains [196] that combine neurons, astrocytes, oligodendrocytes, microglia, and potentially vascular and peripheral immune components. Such models may enable analyses of human cell-cell interactions within the same *in vivo* system. However, increasing cellular complexity will also introduce new challenges in controlling cell composition, developmental timing, reproducibility, and experimental interpretation. Parallel efforts to establish chimeric models in large animals may complement rodent systems by enabling assessment of graft survival, integration, safety, and function across larger anatomical scales and longer experimental periods, although their greater complexity, cost, and ethical considerations will limit widespread implementation.

As these technologies continue to evolve, chimeric CNS models are poised to become increasingly versatile platforms linking human-specific mechanistic studies with therapeutic innovation. Beyond enabling the study of human-specific disease biology, they will accelerate the development of precision regenerative medicine by supporting the evaluation of patient-derived and genetically engineered cells capable not only of replacing vulnerable neural populations but also of delivering therapeutic molecules, modulating the local microenvironment, or correcting disease-associated pathways *in situ*.

## Conclusions

The chimera has become, in modern neuroscience, a powerful experimental platform for studying human brain cells within the physiological environment of the living CNS. Across the major neurodegenerative and demyelinating disorders discussed in this review, chimeric models have revealed human-specific cellular phenotypes and disease mechanisms (Fig. 2). These include cell-autonomous tau pathology and selective neuronal vulnerability in AD, human dopaminergic neurodegeneration and Lewy pathology in PD, mutant huntingtin-driven glial dysfunction in HD, disease-associated astrocyte and microglial programs in ALS, and the remarkable capacity of human oligodendrocyte progenitors to generate compact myelin around host axons in demyelinating disorders. Collectively, these studies highlight a fundamental principle: human brain cells preserve key aspects of their species-specific identity while integrating into, and interacting with, the complex cellular environment of the living CNS.

Beyond providing mechanistic insights, chimeric models have evolved into versatile translational platforms that enable the development and evaluation of regenerative therapeutic strategies while facilitating the identification and validation of novel therapeutic targets (Fig. 2). This translational potential is already beginning to reach the clinic, as illustrated by ongoing trials of hiPSC-derived dopaminergic progenitor transplantation for PD and multiple cell therapy approaches for ALS, which have generally demonstrated favorable safety profiles despite only modest or transient clinical benefits to date.

Looking ahead, continued advances in stem cell engineering, genome editing, multi-lineage humanization, and immune system reconstruction will further increase the biological fidelity and translational relevance of chimeric models. As these technologies mature, they are poised to serve a dual purpose: unraveling the human-specific biology that drives neurodegeneration while accelerating the development of regenerative therapies capable of replacing, repairing, or functionally modifying diseased neural circuits. Once a symbol of the unattainable, the chimera has become a powerful system to uncover human-specific disease mechanisms and to guide the development of regenerative therapies.

## Data Availability

No datasets were generated or analysed during the current study.

## Abbreviations

6-OHDA: 6-Hydroxydopamine
ɑSyn: ɑ-Synuclein
Aβ: Amyloid-β
AD: Alzheimer's disease
ALS: Amyotrophic Lateral Sclerosis
ALSP: Adult-onset leukoencephalopathy with axonal spheroids and pigmented glia
ASPA: Aspartoacylase
CD: Canavan disease
CNS: Central nervous system
DAM: Disease-associated microglia
EAE: Experimental autoimmune encephalomyelitis
EB: Embryoid bodies
fALS: Familial Amyotrophic Lateral Sclerosis
hAPCs: Human astrocyte progenitor cells
HD: Huntington's disease
hESCs: Human embryonic stem cells
hGPCs: Human glial progenitor cells
hiPSCs: Human induced pluripotent stem cells
hNPCs: Human neural progenitor cells
hOL: Human oligodendrocyte
hOPCs: Human oligodendrocyte progenitor cells
hPSC: Human pluripotent stem cell
HRE: Hexanucleotide repeat expansion
HSPG: Heparan sulfate proteoglycan
HTT: Huntingtin
JC: John Cunningham
JHMV: JHM strain of mouse hepatitis virus
mDA: Midbrain dopaminergic
mHTT: Mutant huntingtin
LPC: Lysophosphatidylcholine/lysolecithin
MLD: Metachromatic leukodystrophy
MN: Motor neurons
MPTP: 1-Methyl-4-phenyl-1,2,3,6-tetrahydropyridine
MS: Multiple Sclerosis
MSC: Mesenchymal stem cells
MSNs: Medium spiny neurons
PD: Parkinson's disease
PFFs: Preformed fibrils
PML: Progressive multifocal leukoencephalopathy
QA: Quinolinic acid
RNA-seq: RNA sequencing
sALS: Sporadic Amyotrophic Lateral Sclerosis
SNpc: Substantia nigra pars compacta
TH: Tyrosine hydroxylase
